# Antimicrobial photodynamic inactivation as an alternative approach to inhibit the growth of *Cronobacter sakazakii* by fine-tuning the activity of CpxRA two-component system

**DOI:** 10.3389/fmicb.2022.1063425

**Published:** 2023-01-17

**Authors:** Jinchun Xu, Huangbing Yao, Yali Li, Qiaoming Liao, Xiaoxiao Wan, Lulu Liu, Xiaojing Ma, Han Tao, Hui-Li Wang, Yi Xu

**Affiliations:** School of Food Science and Bioengineering, Hefei University of Technology, Hefei, China

**Keywords:** *Cronobacter sakazakii*, antimicrobial photodynamic inactivation, hypocrellin B, CpxRA, powdered infant formula

## Abstract

*Cronobacter sakazakii* is an opportunistic foodborne pathogen primarily found in powdered infant formula (PIF). To date, it remains challenging to control the growth of this ubiquitous bacterium. Herein, antimicrobial photodynamic inactivation (aPDI) was first employed to inactivate *C. sakazakii*. Through 460 nm light irradiation coupled with hypocrellin B, the survival rate of *C. sakazakii* was diminished by 3~4 log. The photokilling effect was mediated by the attenuated membrane integrity, as evidenced by PI staining. Besides, scanning electron microscopy showed the deformed and aggregated cell cluster, and intracellular ROS was augmented by 2~3 folds when light doses increase. In addition to planktonic cells, the biofilm formation of *C. sakazakii* was also affected, showing an OD_590nm_ decline from 0.85 to 0.25. In terms of molecular aspects, a two-component system called CpxRA, along with their target genes, was deregulated during illumination. Using the knock-out strain of ΔCpxA, the bacterial viability was reduced by 2 log under aPDI, a wider gap than the wildtype strain. Based on the promoted expression of *CpxR* and *OmpC*, aPDI is likely to play its part through attenuating the function of CpxRA-OmpC pathway. Finally, the aPDI system was applied to PIF, and *C. sakazakii* was inactivated under various desiccated or heated storage conditions. Collectively, aPDI serves as an alternative approach to decontaminate *C. sakazakii*, providing a new strategy to reduce the health risks caused by this prevalent foodborne pathogen.

## Introduction

*Cronobacter sakazakii* (formerly known as *Enterobacter sakazakii*) is an emerging foodborne pathogen that can adhere tightly to the surface of packaging materials and cause life-threatening symptoms (Chauhan et al., 2020; Parra-Flores et al., 2021). This species of bacteria has strong propensity of forming biofilm, which constitutes the major virulence of *C. sakazakii* to survive in a broad range of food and food ingredients, such as dairy product, cereal, meats and drinking water (Friedemann, 2007; Harouna et al., 2020; Hong et al., 2022). Of importance, powdered infant formula (PIF) is long considered as the major source for *C. sakazakii*-related risks, while being defined as a category A pathogen of relevance to PIF by FAO/WHO (Gan et al., 2021). PIF contamination can lead to severe health consequences for newborns, exemplified by infantile septicemia, meningitis, necrotizing enterocolitis, etc. (Friedemann, 2009; Chauhan et al., 2020). Based on this situation, developing efficient means to prevent and control *C. sakazakii* offers benefits for food safety and public health.

*Cronobacter sakazakii* is a microbe with high tolerance to desiccation (Barron and Forsythe, 2007), allowing it to persist in PIF. To eliminate it, a range of natural compounds were previously put into practice, such as probiotics, antibiotics, prebiotics, food-grade organic acids, *Chrysanthemum buds* crude extract as well as bacteriocins (Chauhan et al., 2020; Chang et al., 2021; Zheng et al., 2021). In some cases, these substances were used in combination with physical methods to strengthen the bactericidal effect, like sonication, high-hydrostatic pressure, microwave, and UV irradiation (Chauhan et al., 2020; Liao et al., 2021). Nonetheless, some undesired consequences might arise from the use of conventional methods, such as antibiotic resistance, food incompatibility and health/environmental risks (Silva et al., 2018; Rakitin et al., 2022). To resolve this issue, blue light was recently adopted to reduce the survival of *C. sakazakii*, which showed that, a 405 nm-LED irradiation destroyed the microbial biofilm in a significant way. During the process, light illumination lasted for 4 h at a power of 26 mW/cm^2^, decreasing the survival rate by 2.5 log (Huang et al., 2020). While this novel approach did not introduce additional health burden, the antimicrobial efficiency might be further improved by the joint use of photosensitizers (Huang et al., 2020).

Antimicrobial photodynamic inactivation (aPDI) is the application of a photosensitizer (PS), which can be photoactivated with a specific wavelength of light, to generate cytotoxic ROS in the presence of ambient molecular oxygen (Hu et al., 2018; Ma et al., 2022). Photodynamic technique was first developed as an antitumor therapy (Li et al., 2021), and its use in foodborne pathogen control is still in its infancy. aPDI has several advantages over conventional antibacterial methods: firstly, aPDI normally produce a mixture of ROS and singlet oxygen, thus there is a low possibility of triggering bacterial resistance; secondly, aPDI is effective against the unique set of microorganisms, showing apparent microbial specificity; lastly, the antimicrobial effect can be achieved by visible light, reducing the risk of the alternative ultraviolet rays (Su et al., 2011b; Bartolomeu et al., 2016). Compared to the blue light alone, the bactericidal effect is anticipated to be augmented by PS addition, due to the improved energy capture and ROS stimulation (Rapacka-Zdonczyk et al., 2021b). Recently, aPDI exhibited potency in inactivating some food-derived microorganisms, like *Alicyclobacillus acidoterrestris*, *Staphylococcus aureus*, *Escherichia coli*., etc. (do Prado-Silva et al., 2020). Yet, it remains unknown if aPDI has inhibitory effect on *C. sakazakii* in a food-sterilizing practice.

Among the natural photosensitizers, hypocrellin received considerable attention owing to its easy preparation, high ROS yields, low toxicity and rapid metabolism *in vivo* (Al-Mutairi et al., 2018; Romero et al., 2021). As a natural product from traditional Chinese medicine, hypocrellins were believed harmless to human body, thus having potential to be used as safe and stable additives in food industry (Su et al., 2011a), which consitutes a plausible rationale to test its antimicrobial activity in food-related processes. Hypocrellin A is able to photo-inactivate a wide spectrum of microbes including *S. aureus*, *Bacillus subtilis*, *Salmonella typhimurium* and *Candida albicans* (Ma et al., 2004; Yang et al., 2019). As a derivative, the photodynamic activity of hypocrellin B (HB) was less understood. In an example, this photosensitizer was found potent in curbing *S. aureus* by doing harm to membrane permeability (Jiang et al., 2013). Moreover, our previous findings discovered the synergistic photodynamic action with curcumin or titanium nanoparticles against *S. aureus*, leading to the remarkable bacterial mortality (Li et al., 2021; Wan et al., 2022). Although the gram-positive bacteria generally displays higher susceptibility to aPDI than the gram-negative counterparts on account of their different cell envelope structures (Rapacka-Zdonczyk et al., 2021a), we still need to try to transfer this HB-mediated regimen to *C. sakazakii*, to explore if this new aPDI approach could be used as an alternative to fight against this foodborne pathogen.

In this study, the inhibitory effect of the HB-mediated aPDI on *C. sakazakii* was investigated, followed by the causative mechanisms specifying membrane permeability, cell envelope morphology, ROS functioning as well as molecular insight. Subsequently, a CpxA-knock out strain was established to interrogate roles of CpxRA in the studied oxidative stress, owing to its apparent changes upon aPDI. Moreover, aPDI was potent in decontaminating PIF under various storage conditions, which promises to apply aPDI in the control of *C. sakazakii* during food preservation.

## Materials and methods

### Bacterial strain and growth conditions

*Cronobacter sakazakii* (ATCC 29544) used in this study was purchased from Beijing BeNa Culture Collection (Beijing, China). Strains were maintained at −80°C and the working culture was obtained following the instructions of the supplier. The frozen stock culture was transferred into a 5-mL sterile tryptic soy broth (TSB; Land Bridge Technology, Beijing, China), and the overnight culture was inoculated to a fresh TSB medium at a ratio of 1% (v/v) and incubated at 37°C with agitation, till cell concentration reached 10^8^~10^9^ CFU/mL. The growth curve was depicted by measuring OD_600nm_ values at different growth timepoints. *E. coli* β2155 was derived from the strain repository of this lab and grown at 37°C under aeration for cloning of recombinant vectors.

### Photosensitizer and light source

Hypocrellin B (HB) purchased from Yua-nye Biotech (Shanghai, China) was first dissolved in dimethyl sulphoxide (DMSO) at a concentration of 1 mM and stored at 4°C in the dark before the photosensitizer was used for further experiments. The light source used here was LED with a wavelength of 460 nm and a power adjusted to 50.8 mW/cm^2^. The LED system (CREE; Durham, NC, United States) was set up in a closed chamber to avoid the interference of ambient visible light. The optical output power was calibrated by a laser power meter (VLP-2000, Beijing, China). The 460 nm LED was attached to an aluminum heat sink to dissipate the heat generated.

### Antimicrobial photodynamic inactivation treatment and bacterial enumeration

Antimicrobial photodynamic inactivation treatment was performed as previously described (Demidova and Hamblin, 2005). The bacteria at a mid-log phase were centrifuged to remove the supernatant and incubated in PBS with varying concentrations of photosensitizers (0, 10, 15, 20, 25, and 30 μM for HB) in a 37°C incubator under dark environment for 30 min, reaching an optical density of 0.8. The bacterial preparation (1 mL) was added to a 24-well plate and irradiated with 460 nm-LED light at room temperature. The light intensity ranged from 0 to 60 J/cm^2^ at a power density of 50.8 mW/cm^2^. All aPDI experiments were compared with three control groups: blank control, light only or photosensitizer only. All samples were serially diluted with sterile PBS (10^−1^~10^−6^). To determine the appropriate dilution factor, 1 μL of aliquot with various dilution was subjected to TSB solid media (supplemented with 1.3% agar powder) for dot plating. Aliquots of 50 μL were plated in TSB media for enumeration of viable cells. The plates were incubated in a 37°C incubator for 24 h and the emerging colonies were counted. The survival fraction of *C. sakazakii* was then calculated relative to the control group.

### Propidium iodide and double staining

Propidium iodide (PI) and LIVE/DEAD^®^ BacLight^™^ Bacterial Viability Kit (Molecular Probes, Invitrogen, Carlsbad, United States) were used here to detect bacterial membrane integrity and cell viability, respectively. Double-staining performs well in deciphering live/dead ratio, while PI alone could better detect membrane penetrance without interference of other dyes. Following aPDI exposure, the bacteria were centrifuged at 5,000 g for 3 min and washed twice with sterile PBS. The harvested cells were then incubated with PI (3 μL per mL) in the dark at 37°C for 15~20 min. After washing twice with PBS to remove excess dye, the suspension was then transferred to a glass slide for fluorescence microscopy examination (ECLIPSE Ti2-U, Nikon, Tokyo, Japan) using a 590 nm bandpass filter. The fluorescence intensity of each image was quantified by Image J software (NIH, Bethesda, MD, United States). Double staining results are expressed by the ratio of live (green) and dead bacteria (red; Zou et al., 2020).

### Intracellular DNA leakage

The bacterial cells were collected by a centrifugation at 8,000 g for 3 min after aPDI treatment. The supernatant preparations were filtered with a 0.22 μm microporous membrane. The concentration of nucleic acids was then determined using a microplate spectrophotometer (Infinite 200 Professional, Tecan, Mannedorf, Switzerland) by OD_260nm_ values, according to the manufacturer’s instructions.

### Scanning electron microscopy

SEM was performed as described previously (Jan et al., 2019). In brief, *C. sakazakii* cells were collected, added to the fixative buffer for 2 h at room temperature, and then fixed at 4°C overnight. The sample was then sequentially washed 3 times and eluted with 50, 70, 80, and 90% ethanol, respectively. After another round of centrifugation, ethanol was added to the pellet and incubated for 30 min. Upon ethanol dehydration, the samples were then freeze-dried, coated with gold and observed using the scanning electron microscope (X650, Hitachi, Tokyo, Japan).

### Intracellular ROS measurement

After aPDI treatment, 3 μL DCFH-DA (2′, 7′-dichlorofluorescein diacetate) fluorescent dye (Sigma-Aldrich, Shanghai, China) was added to *C. sakazakii* suspension and incubated at 37°C for 30 min. The excess dye was then washed off and the cells were transferred to a glass slide. Fluorescence signals were observed with fluorescence microscope (Nikon, Tokyo, Japan), and intracellular ROS levels were analyzed using Image J software.

### Enzymatic activity assay

The aPDI-treated (30 μM HB under irradiation of various light doses) *C. sakazakii* was centrifuged at 3,000 g for 7 min to remove the supernatant and resuspended in an equal volume of sterile PBS. The enzymatic activity of CAT (catalase) was determined with a CAT detection kit (A007-1-1, Jiancheng, Nanjing, China) according to the manufacturer’s instructions. Absorbance at 405 nm was then measured using a microplate spectrometer (infinite 200 pro, Tecan, Mannedorf, Switzerland).

### Biofilm quantification

Biofilm quantification was performed by crystal violet (CV) assay. The aPDI-treated *C. sakazakii* was diluted in TSB and 200 μL of aliquot was transferred to 96-well plates (Corning Life Sciences, Acton, MA) for biofilm formation. The incubation was performed in a 37°C incubator for various length of time (12 h, 24 h, 36 h, and 48 h), to indicate different stages of biofilm dynamics. After incubation, the culture was washed twice and fixed at 60°C for 1 h. The biofilms were then stained with 200 μL of 0.1% crystal violet (CV) for 20 min in the dark, washing 3 times with PBS to remove excess dye. The stained biofilms were then imaged using fluorescence microscope (ECLIPSE Ti2, Nikon, Tokyo, Japan). The OD value of each well was determined at a wavelength of 590 nm using microplate spectrophotometer (Infinite 200 PRO, Tecan, Mannedorf, Switzerland), following biofilm dissolution in 33% acetic acid.

### qPCR analysis

Total RNAs were extracted from aPDI-treated *C. sakazakii* using the RNeasy Protect Bacteria Mini Kit (Qiagen, Shanghai, China). Subsequently, the reverse transcription reaction was performed using First-Stand cDNA Synthesis SuperMix (Transgen Biotech, Beijing, China) according to the manufacturer’s instructions, to generate cDNA and stored at-20°C until further experiments. qPCR was performed based on cDNA templates using LightCycle96 Q-PCR (Roche, Basel, Switzerland). The relative transcription levels of the objective genes were detected by real-time quantitative reverse transcription PCR, with primers listed in Table 1. Relative expression of individual genes was quantified using 16S rRNA as an internal control. The PCR process consisted of pre-denaturation and 40 cycles of amplification (denaturation at 95°C for 30 s and annealing at 55°C for 30 s).

**Table 1:** Primers used in this study.

Primer	Sequence	Target gene
FliCF	CTTACAGCGTATCCGTGAGC	*FliC*
FliCR	GCCGTTGAAGTTAGCACCA	
FliKF	GTTGCCCAATGTTGTGCTTA	*FliK*
FliKR	ATTTCGCCGTTGGTCAGTT	
FlgKF	CGTTCTGGGGCAGTCTAACA	*FlgK*
FlgKR	GACCATATCGTCGATTTTCG	
FliHF	ACCTGATTAAGCAGATCCAGAC	*FliH*
FliHR	TTCATCGGCGGAGACTTTG	
FlgIF	GATTCCTGCTGTTGCTCGTC	*FlgI*
FlgIR	TCAGGCTCTGGGTGGTAAA	
OmpCF	CTACCGTAACACCGACTTCTTC	*OmpC*
OmpCR	GCTGCCAGGTAAATGTTGTT	
CpxRF	GCGGATGATTACCTGCCAA	*CpxR*
CpxRR	GCCGTTATCGCTGTTCTGC	
CpxAF	AGGGCACGATGATTGAGCA	*CpxA*
CpxAR	CTGACCGATAAAGTTGCGAATG	
CpxPF	GCTGGTCACCGCAGAAAAT	*CpxP*
CpxPR	GCGTTGCTGATGTTTCTTGTTC	
CpxA5F	TCGAGGCTGAGCTGGCGGGCGACC	Upstream arm of *CpxA*
CpxA5R	CAGCATTGTGACGATCAGCAGTAGC	
CpxA3F	CAGCATGATTAACGATCTGCTGGTG	Downstream arm of *CpxA*
CpxA3R	GATTCGCGGTCGCGCGCCTCGTCA	
CpxAVF	ATGATAGGCAGCCTTACCGCCCGCA	*CpxA*
CpxAVR	TCAGGATCGGTGATAAAGCGGCAAC	
926F	AAACTCAAAKGAATTGACGG	*16S rRNA*
1062R	CTCACRRCACGAGCTGAC	

### Mutant strain construction and genetic manipulation

Suicide plasmid was constructed by ligating upstream and downstream fragments of *CpxA* into pCVD442, with the aid of Gibson cloning. The homologous arms were amplified by PCR from *C. sakazakii* ATCC 29544 genomic DNA using primer pairs CpxA5F/R and CpxA3F/R, respectively. The recombinant plasmids were then introduced by electroporation into *E. coli* strain β2155. After ligation, the resultant plasmid was transformed into the recipient *C. sakazakii* strain by conjugation. The deletion mutants were selected from sequencing the trans formant colonies, and the isolated genomic DNA was subjected to PCR verification by the primer pair CpxAVF/R (Table 1).

### Photodynamic treatment on powdered infant formula

The PIF trial was referenced to a previous study (Zheng et al., 2021), with some modifications: the *C. sakazakii* culture at a mid-log phase was centrifuged at 8,000 g for 3 min at 4°C, washed twice, mixed with powdered infant formula (PIF, Good Start Soy, Nestle, Beijing, China) with a ratio of 1:20 (w/w). The used PIF contains 12.5% protein, 8.1% polyunsaturated fatty acid and 55.6% carbohydrate (w/w), along with other ingredients. The mixture was incubated without a lid in a sterile incubator at 37°C for 2 day to air-dry before being transferred to a 24-well plate for aPDI treatment. The plate counting was then carried out following aPDI exposure to check the validity of PIF decontamination: after samples were withdrawn and reconstituted in PBS, appropriate dilutions were plated on TSB medium, and the bacterial colonies were then counted after the growth of 24 h at 37°C.

In aPDI-treated PIF samples, pH values were determined to assess food quality by a pH meter (Leitz, Shanghai, China), which was pre-calibrated with standard solution before use.

*Cronobacter sakazakii* can survive under a range of environmental conditions, represented by desiccation and high temperature, which contributes to its prevalence in PIF. Hence, to investigate effect of aPDI on the bacterial resistance to desiccation, the aPDI-treated PIF was transferred to 24-well plates and stored without a lid in a 37°C sterile incubator pre-treated with air-drying for 2 weeks (Amalaradjou and Venkitanarayanan, 2011). The samples were collected at 0, 1, 2, 4, 7, and 14 day during storage, reconstituted in PBS, and plated in TSB medium for counting. The experimental results are expressed as log_10_ (*N*/N0). *N* is the microbial population after drying (CFU/g), while N0 is the initial cell number before drying (CFU/g).

The ability of *C. sakazakii* in PIF to survive heat stress was investigated at 45°C, 50°C, and 55°C. The 1.5 g formula was reconstituted in 10 mL sterile distilled water, and 1 mL aliquot was pipetted into 1.5 mL Eppendorf tube, which was subsequently incubated in a temperature-controlled water bath. At various time intervals (0, 20, 40, 60, and 90 min), PIF preparations were collected and subjected for plate counting of viable cells. The data was obtained using the same formula as desiccation trials.

### Statistical analysis

Values are expressed as mean ± SEM (standard error of mean) from at least three independent biological replicates. Statistical analysis was performed using SPSS software (version 19.0, IBM, NY, United States). Independent-sample *t*-test was used to indicate two group comparisons, and one-way analysis of variance (ANOVA) with *post hoc* test was adopted to perform multiple comparisons. *p* < 0.05 indicates a significant difference among groups.

## Results

### Inhibitory effect of antimicrobial photodynamic inactivation on *Cronobacter sakazakii*

**Figure 1:**
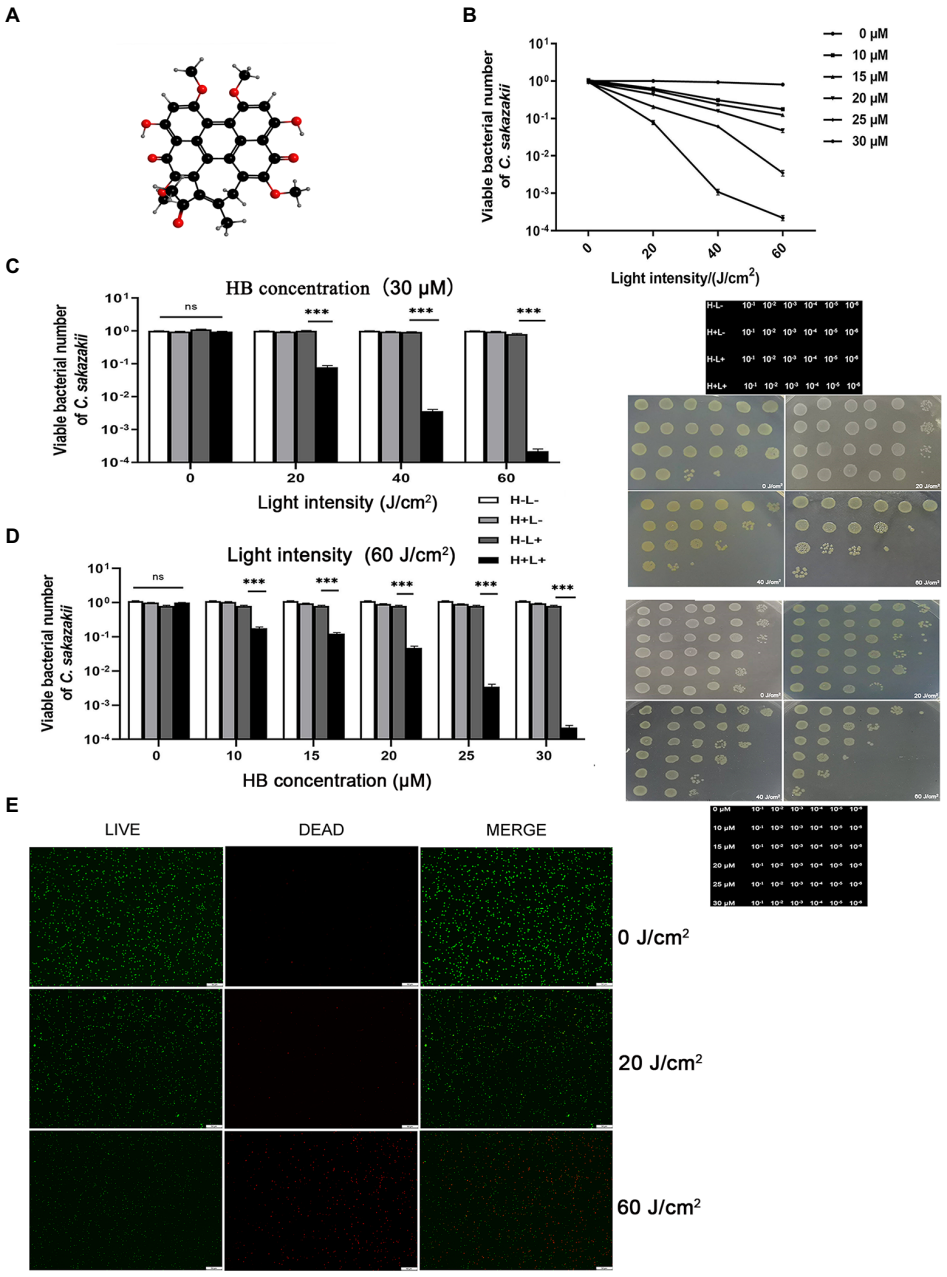
Inhibitory activity of antimicrobial photodynamic inactivation on *Cronobacter sakazakii*. The aPDI based on hypocrellin B **(A)** was used to control the growth of *C. sakazakii* ATCC29544. The survival rate **(B)** of this strain was calculated relative to the dark control, according to the plate counting data. The photodynamic conditions were set as 10, 15, 20, 25, 30 μM for HB, and 20, 40, 60 J/cm^2^ for light intensity, respectively. The influence of light intensity **(C)** and HB concentration **(D)** on the bacterial survival were then plotted in bar charts, whereas three control groups (blank, light only and HB only) were designed to fully indicate the aPDI effect. The representative dot plating graphs as well as the layout on the medium plate were also shown. In (C), 30 μM HB was used, and in **(D)**, 60 J/cm^2^ light was used. To validate this effect, the LIVE/DEAD double staining was carried out **(E)**, and green (live), red (dead) and merged images were obtained from fluorescence microscopy. This assay was performed in cells treated with 30 μM HB. The scale bar represents 50 μm. H-L-, blank control; H+L-, single HB-treated group; H-L+, single light-treated group; H+L+, the aPDI-treated group. Values are the mean ± SEM (standard error of mean) from 6 replicates. ^*^p < 0.05, ^**^p < 0.01, ^***^p < 0.001.

The inhibitory effect of aPDI on the survival rate of *C. sakazakii* was first evaluated. The aPDI was carried out based on the energy conversion activity of HB, a natural pigment with perylenequinone structure (Figure 1A). The growth curve of *C. sakazakii* was determined (Supplementary Figure S1) and cells in the mid-log phase were harvested for aPDI test. The doses of photosensitizer were set as 10, 15, 20, 25, and 30 μM, respectively and light intensities of 460-nm LED were adjusted to 20, 40, or 60 J/cm^2^. Under these conditions, the survival rate of *C. sakazakii* was sharply decreased, reaching a maximum 3.6 log when 30 μM HB was irradiated by 60 J/cm^2^ of blue light (Figures 1B–D). Besides, it’s obvious that the aPDI efficacy was highly dependent on the HB dosage and light intensity used. The representative dot-plating was shown in Figures 1C,D. The bacterial injury was not induced by DMSO, as the solvent alone did not display antimicrobial effect (Supplementary Figure S2). Besides, no dark toxicity of HB was detected in the studied settings (Figures 1B–D).

We next used LIVE/DEAD double staining to consolidate the efficacy of aPDI. 30 μM HB was selected for the subsequent experiments due to the prominent photokilling effect. As evidenced by Figure 1E, the ratio of live/dead *C. sakazakii* cells was diminished at an irradiation of 20 J/cm^2^, and a rigorous condition (60 J/cm^2^) augmented the inhibitory impact. To summarize, the HB-based aPDI could inactivate *C. sakazakii* in a significant way.

### Effect of antimicrobial photodynamic inactivation on the membrane integrity of *Cronobacter sakazakii*

**Figure 2:**
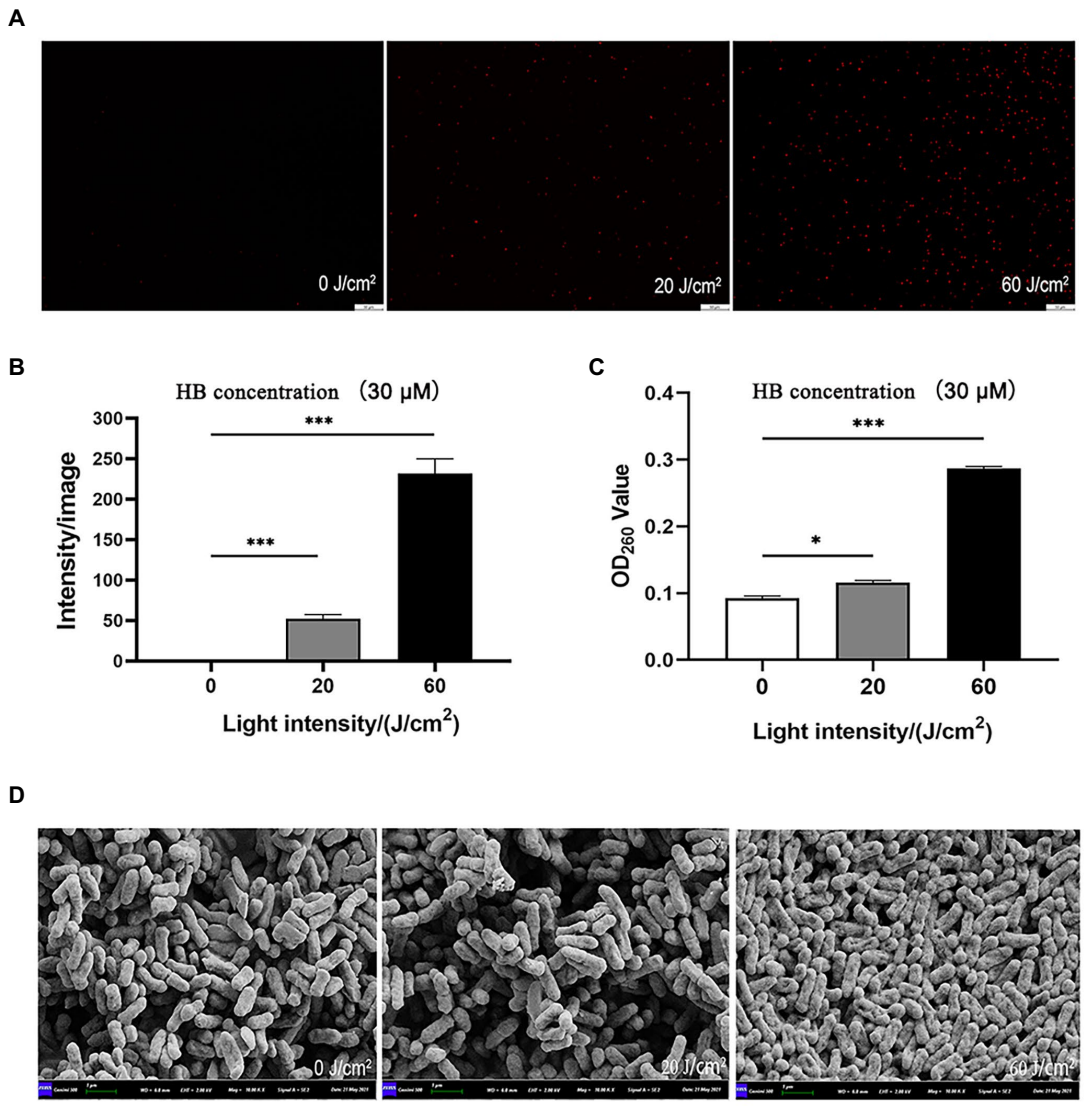
Effect of aPDI on the membrane integrity of *C. sakazakii*. The aPDI-treated bacterial cells were subjected to PI staining. Representative fluorescence microscopy images **(A)**, as well as their quantification **(B)** were shown in cells treated with 30 μM HB photo-activated by various light doses. The scale bar represents 50 μM. In addition, DNA leakage **(C)** was evaluated by measuring OD_260nm_ of extracellular substances, and cell surface structure was profiled by scanning electron microscopy **(D)**. The bar represents 1 μm. Values are the mean ± SEM (standard error of mean) from 5~6 replicates. ^*^p < 0.05, ^**^p < 0.01, ^***^p < 0.001.

In order to explore mechanisms underlying the aPDI-induced injury of *C. sakazakii*, PI staining was performed to examine the bacterial membrane integrity. According to the results, the membrane integrity was severely damaged by aPDI (Figures 2A,B). This phenomenon was further consolidated by the enhanced DNA leakage (Figure 2C), which is an iconic molecular event of membrane damage.

Membrane damage is closely associated with surface morphological changes (Hu et al., 2018). According to SEM graphs, after aPDI treatment, the anatomical features of *C. sakazakii* was prone to a series of changes, manifested by distorted, wrinkled and shrunken surface morphology (Figure 2D). Of note, bacterial cells tended to auto-aggregate as light intensity increased, a process involving the attachment of the released substances. Taken together, aPDI has detrimental effect on the membrane integrity of *C. sakazakii*.

### ROS stimulated by antimicrobial photodynamic inactivation on *Cronobacter sakazakii*

**Figure 3:**
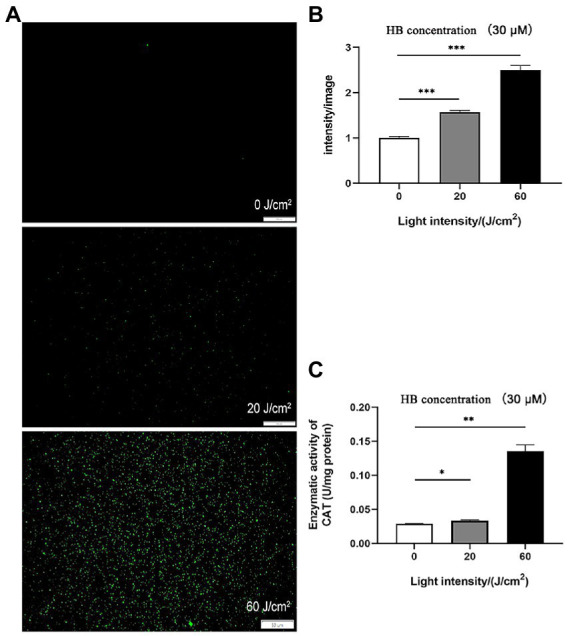
ROS stimulated by aPDI on *C. sakazakii*. Intracellular ROS was analyzed using DCFH-DA staining in *C. sakazakii* cells, in response to aPDI with 30 μM HB irradiated by various light doses. In addition to representative microscopy images **(A)**, fluorescence intensity **(B)** was quantified using Image J software. Scale bar represents 50 μm. In addition, enzymatic activity of catalase **(C)** was also examined to assess the bacterial antioxidative response. CAT, catalase. Values are the mean ± SEM (standard error of mean) from 3~4 replicates. ^*^p < 0.05, ^**^p < 0.01, ^***^p < 0.001.

Antimicrobial photodynamic inactivation normally acts by generating a unique combination of ROS molecules. To test if this is the causative agent of *C. sakazakii* inactivation, we quantified ROS *in vivo via* DCFH-DA staining. As shown in Figures 3A,B, intracellular ROS was significantly enhanced by aPDI treatment, with the alteration of 2.5 folds under the irradiation of 60 J/cm^2^. This result might implicate ROS generation in the resulting bacterial death. Interestingly, the imposed oxidative stress augmented the enzymatic activity of bacterial catalase (Figure 3C). This change might be attributed to the intrinsic antioxidative response of *C. sakazakii*, which, however, did not prevent the light-induced accumulation of ROS. ROS was thought to attack proteins, lipids and nucleic acids inside the microbial cells (Hu et al., 2018). Thus, aPDI promotes the generation of ROS, which might play part in the impairment of *C. sakazakii* cells.

### Effect of antimicrobial photodynamic inactivation on the biofilm of *Cronobacter sakazakii*

**Figure 4:**
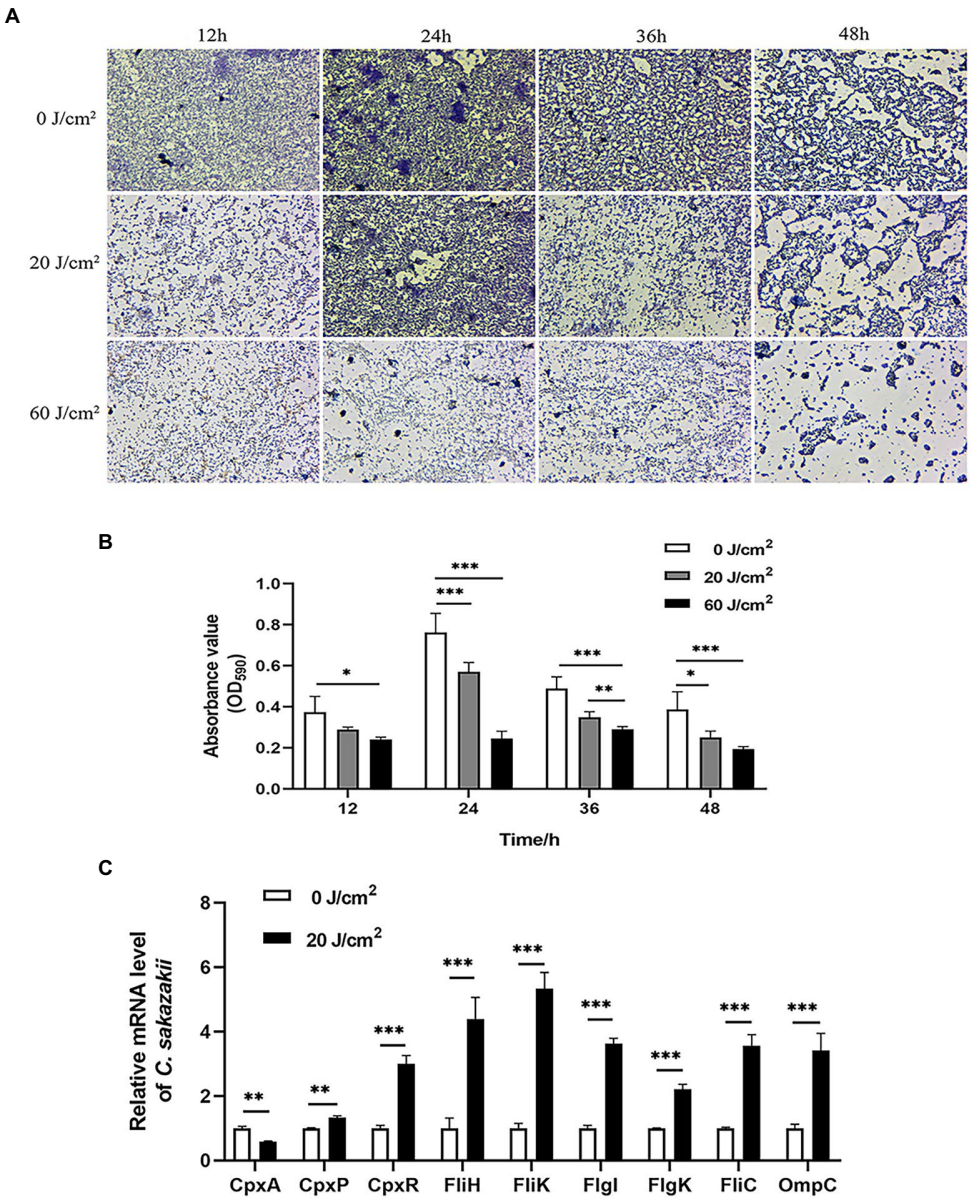
Effect of aPDI on the biofilm of *C. sakazakii*. *C. sakazakii* was treated with HB illuminated by 460-nm LED (20 or 60 J/cm^2^) before crystal violet staining. OD_590nm_ measurements of CV-stained biofilms grown on 96-well plates **(A)** were performed at various stages of biofilm formation (12 h, 24 h, 36 h, and 48 h) **(B)**. The representative microscopy images were shown to indicate the biofilm status under aPDI-induced pressure. To study molecular changes with relevance to biofilm disruption, qPCR was performed to examine mRNA levels of CpxRA TCS as well as flagella-and porin-encoding genes **(C)**. The aPDI was set as a mild photokilling condition of 20 J/cm^2^ light-30 μM HB, to better reflect the stress responsiveness of *C. sakazakii*. Values are the mean ± SEM (standard error of mean) from 4~6 replicates. ^*^p < 0.05, ^**^p < 0.01, ^***^p < 0.001.

*Cronobacter sakazakii* can form biofilms by adhering to the surface of materials, which allows it to persist in tough environments (Xu et al., 2021). So we next examined the effect of aPDI treatment on the assembled biofilms. The fluorescence microscopy showed that the photo-activated HB significantly disrupted the biofilm structure at various stages, which were observed at 12 h, 24 h, 36 h, and 48 h, respectively, (Figures 4A,B). It was seen from the graphs that the biofilm tended to be dispersed and disintegrated by the continued light irradiation, suggesting that aPDI was able to attack both planktonic and biofilm cells of *C. sakazakii*.

CpxRA signaling plays important roles in controlling sensing and adhesion of *E. coli* (Otto and Silhavy, 2002), thus this TCS system has relevance to biofilm formation. To further inspect the molecular mechanisms underlying biofilm disruption of *C. sakazakii*, the expression levels of *CpxA*, *CpxR*, and *CpxP* were measured in response to aPDI. According to the results, this treatment reduced the mRNA transcripts of *CpxA* while upregulating *CpxR* and *CpxP* (Figure 4C), which may indicate that the CpxRA TCS was deregulated by the imposed oxidative stress. By profiling the CpxRA-targeting genes, some flagella-related genes were found increased, in parallel with an outer membrane porin, *OmpC* (Figure 4C), suggesting that CpxRA pathway was aberrantly stimulated by aPDI. In summary, aPDI has deleterious impact on the biofilm of *C. sakazakii*.

### Effect of antimicrobial photodynamic inactivation on the CpxA-knockout mutant

**Figure 5:**
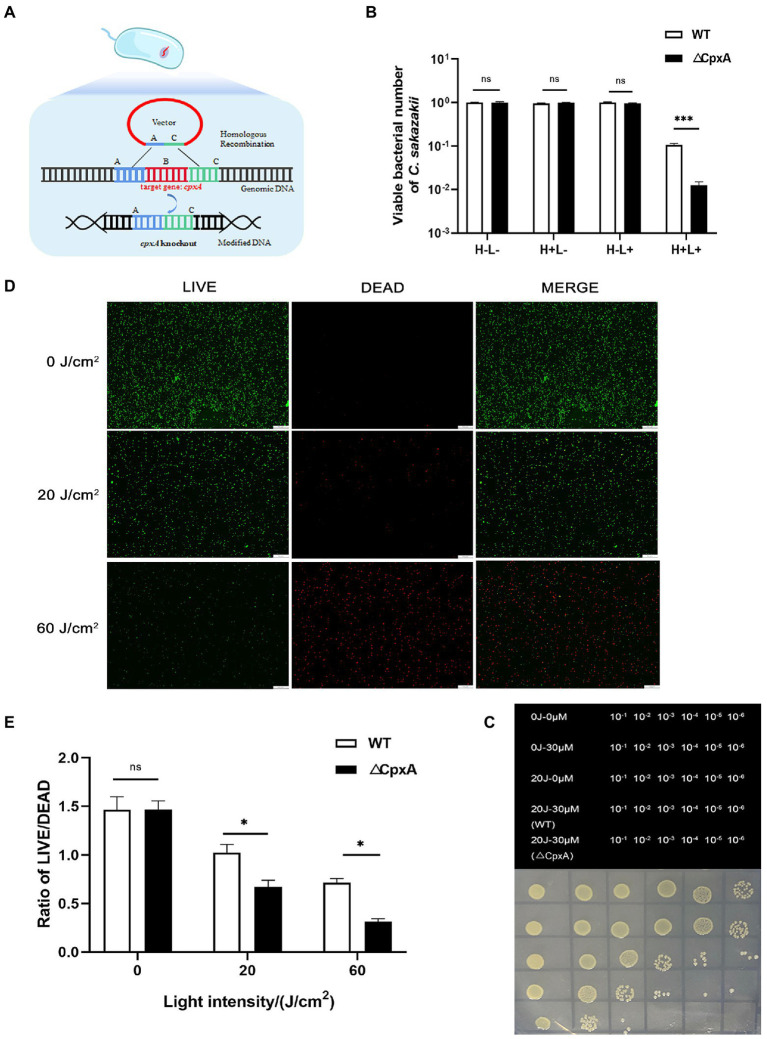
Effect of aPDI on the CpxA-knockout mutant. ΔCpxA mutant was constructed by means of suicide plasmid-mediated recombination **(A)**. The survival rates **(B)** of wildtype and ΔCpxA were compared under the condition of 20 J/cm^2^ light-30 μM HB. The representative dot-plating graphs were also shown as categorized by various dilutions **(C)**. Three control groups (blank, light only and HB only) were designed to indicate the aPDI effect. To validate this effect, the LIVE/DEAD double staining of both strains were performed, and green (live), red (dead) and merged images were obtained from fluorescence microscopy **(D)**. The scale bar represents 50 μm. The quantification was conducted using Image J software **(E)**. H-L-, blank control; H+L-, single HB-treated group; H-L+, single light-treated group; H+L+, the aPDI-treated group. Values are the mean ± SEM (standard error of mean) from 4~6 replicates. ^*^p < 0.05, ^**^p < 0.01, ^***^p < 0.001.

In order to investigate if CpxRA is implicated in the aPDI-mediated removal of *C. sakazakii*, the mutant strain, ΔCpxA, was established by the homologous recombination (Figure 5A), and its growth curve was shown in Supplementary Figure S1: no evident changes were observed compared to wildtype strain. The mutant strain was susceptible to aPDI treatment, as demonstrated by the addition of 30 μM HB excited by 20 J/cm^2^ of illumination (Figures 5B,C). Of note, compared to wildtype strain (0.9 log), the viability of the mutant was curbed to a larger extent (2.0 log), suggesting that CpxA abrogation diminished the antioxidative capacity of *C. sakazakii* under the studied context. This finding was then consolidated by the LIVE/DEAD double staining (Figures 5D,E).

**Figure 6:**
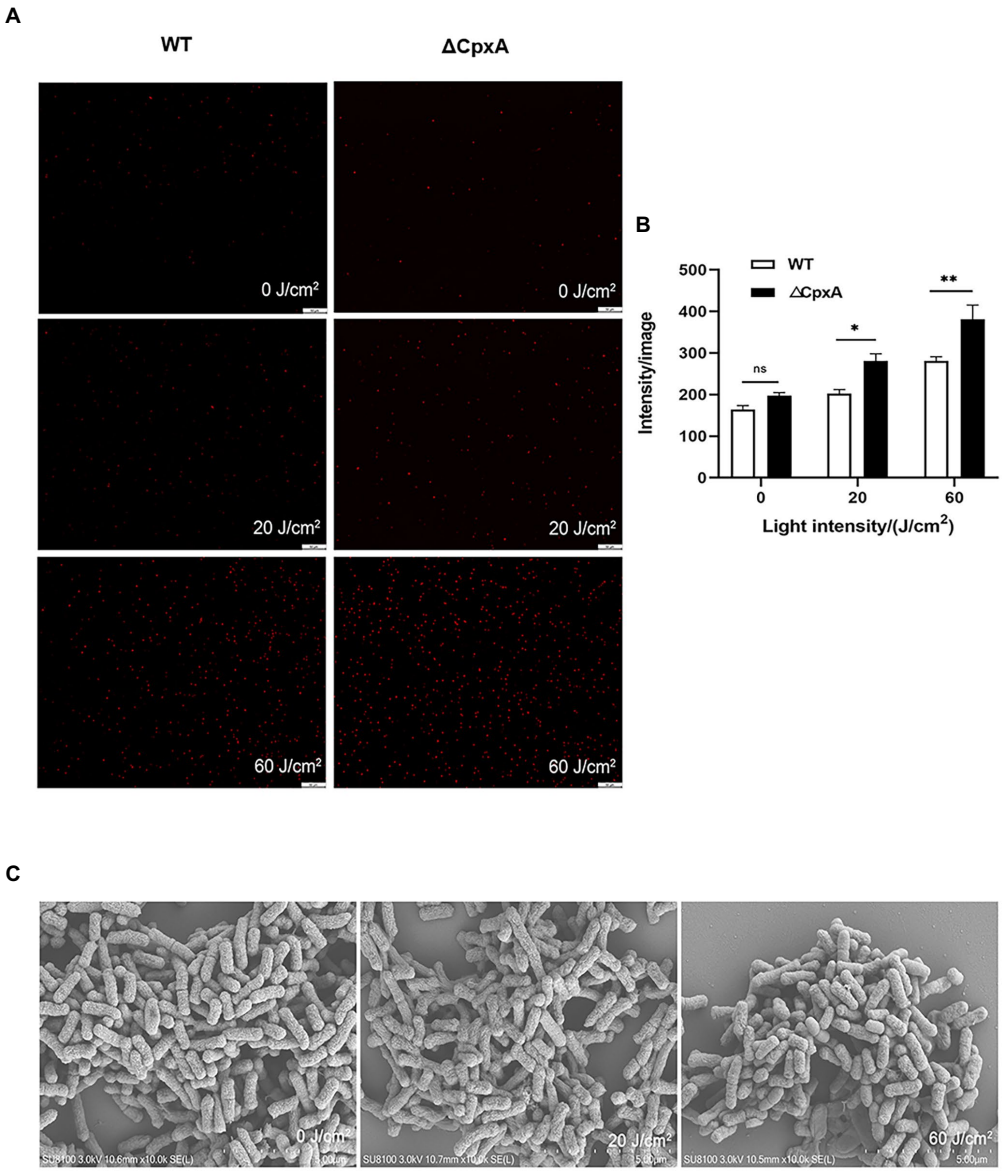
Effect of aPDI on the membrane integrity of ΔCpxA. The aPDI-treated wildtype and ΔCpxA were subjected to PI staining. Representative fluorescence microscopy images **(A)**, as well as their quantification **(B)** were shown in cells treated with 30 μM HB photo-activated by various light doses (20 J/cm^2^, 60 J/cm^2^). The scale bar represents 50 μM. In addition, anatomical features of *C. sakazakii* were observed through SEM with a scale bar set as 1 μm **(C)**. Values are the mean ± SEM (standard error of mean) from 4 replicates. ^*^p < 0.05, ^**^p < 0.01, ^***^p < 0.001, ns, *p* > 0.05.

Considering membrane integrity, PI staining indicated an aggravated permeability due to the deletion of CpxA (Figures 6A,B). In line with the hampered cell envelope morphology (Figure 6C), it might deduce that CpxA plays pivotal roles in modulating the aPDI potency against *C. sakazakii.* Moreover, as unveiled by DCFH-DA staining, an increasing amount of ROS (3 folds) was produced as irradiation prolonged (Supplementary Figures S3A,B).

**Figure 7:**
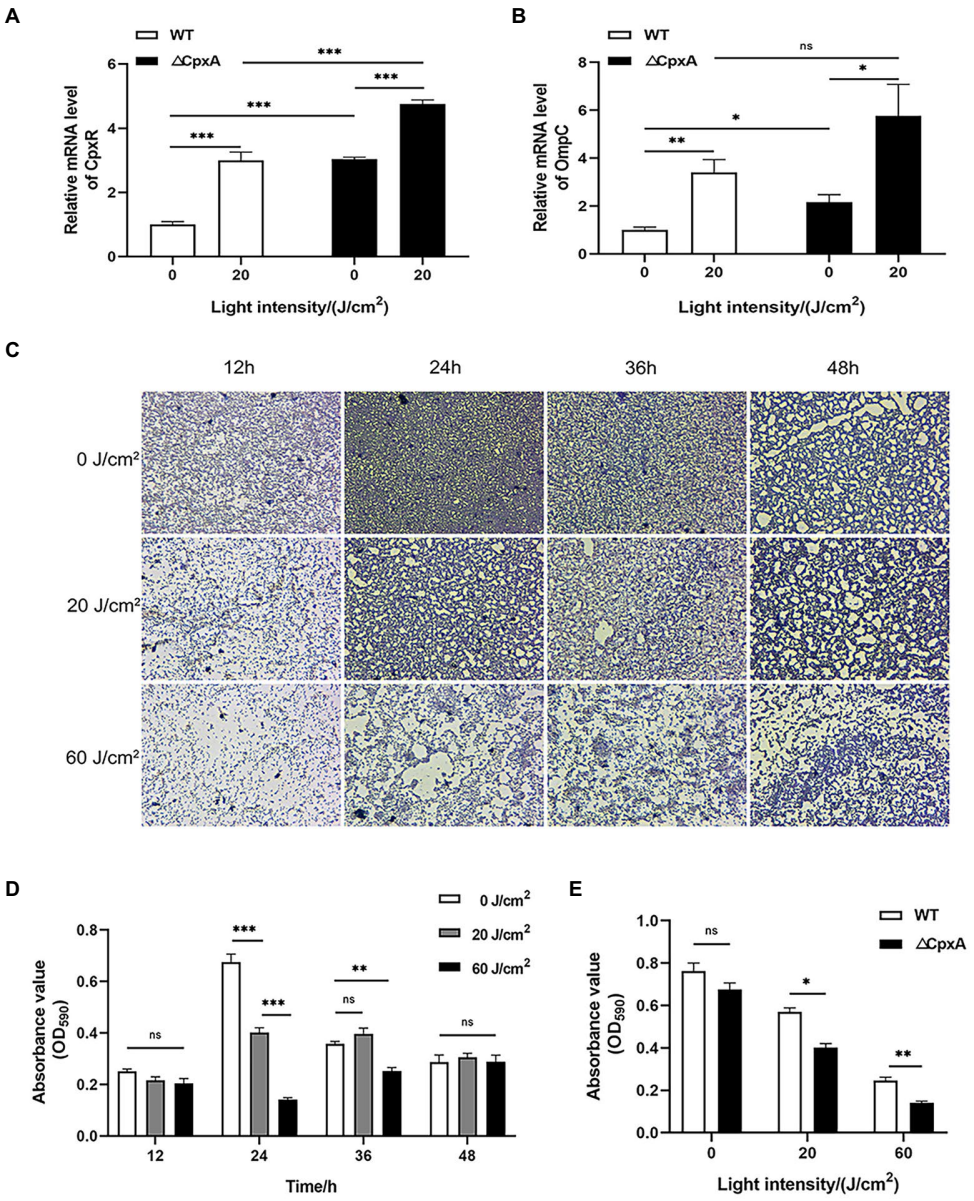
Effect of aPDI on the biofilm formed by ΔCpxA. qPCR was first performed to examine mRNA levels of *CpxR* **(A)** and *OmpC* **(B)**, in response to CpxA deletion as well as aPDI exposure. ΔCpxA strain was treated with HB activated by 460-nm LED illumination (20 or 60 J/cm^2^) before crystal violet staining. OD_590nm_ measurements of CV-stained biofilms grown on 96-well plates **(C)** were performed at the various stages of biofilm formation (12 h, 24 h, 36 h, and 48 h) **(D)**. The representative microscopy images were shown to indicate the biofilm status under aPDI-induced pressure. In addition, the comparisons between wildtype and mutant were performed with respect to their biofilm status at a growth of 24 h **(E)**. Values are the mean ± SEM (standard error of mean) from 4~6 replicates. ^*^p < 0.05, ^**^p < 0.01, ^***^p < 0.001, ns, *p* > 0.05.

By determining the expression profiles, the mRNA level of *CpxR* was upregulated in ΔCpxA, indicating the negative mode of interaction between the molecular partners (Figure 7A). In addition, *OmpC* expression was also promoted upon *CpxA* deletion, and this trend was reinforced by the addition of light-activated HB (Figure 7B). This data might implicate a CpxRA-OmpC pathway in the studied sterilization course. In the event of biofilm formation (Figures 7C–E), the ΔCpxA biofilm displayed a more dispersed status, a proof that CpxA deletion rendered *C. sakazakii* prone to the oxidative stress brought by aPDI in either planktonic or biofilm forms. These results demonstrate that CpxRA modulates the effect of aPDI on *C. sakazakii*.

### Effect of antimicrobial photodynamic inactivation on PIF contaminated by *Cronobacter sakazakii*

**Figure 8:**
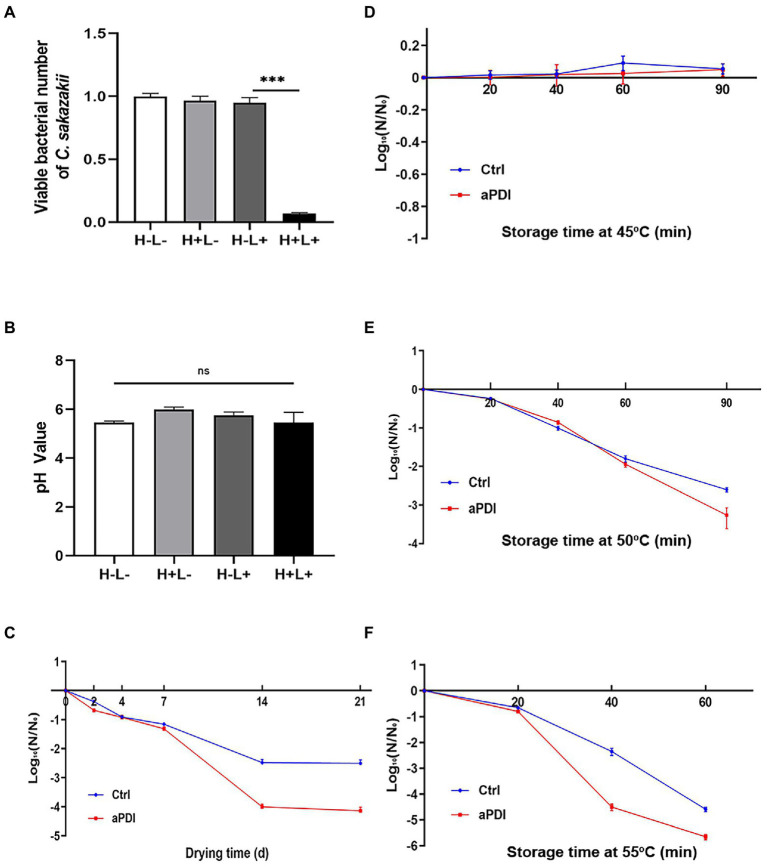
Effect of aPDI on PIF contaminated by *C. sakazakii*. *C. sakazakii* was first inoculated into powdered infant formula (PIF) in a ratio of 1:20 (w/w) to establish a contamination model. The contaminated PIF was then transferred to a 24-well plate for aPDI sterilization. The viable cells were subjected to plate counting in the experimental and controlled groups **(A)**. The food quality upon treatment was assessed by measuring pH values of infant formula **(B)**. The bacterial resistance to desiccation was then evaluated by incubating the aPDI-treated PIF in an air-drying chamber. The samples were collected from various length of days (2, 4, 7, 14, and 21), and subjected to viable cell enumeration **(C)**. The results are expressed as log_10_ (N/N0). *N* is the microbial population after drying (CFU/g), while N0 is the initial cell number before drying (CFU/g). The similar procedure was applied to heated stress trials, wherein varying temperatures, like 45°C **(D)**, 50°C **(E)** and 55°C **(F)**, were designed to study the survival rate of *C. sakazakii* in the reconstituted PIF. Samples were collected in the time intervals of 20, 40, 60, and/or 90 min, respectively. Values are the mean ± SEM (standard error of mean) from 4 replicates. ^*^p < 0.05, ^**^p < 0.01, ^***^p < 0.001, ns, *p* > 0.05.

*Cronobacter sakazakii* is a well-defined foodborne pathogen contaminating PIF (Zhang et al., 2020). To this end, a laboratory PIF model was constructed to investigate the validity of aPDI in reducing the risk of *C. sakazakii* infection. PIF was first inoculated with *C. sakazakii* at a mid-log stage, mixed with hypocrellin B and then subjected to 460-nm LED illumination. After this transient manipulation, the contaminated PIF was placed at room temperature for a variable length of time, followed by bacterial enumeration. The results showed that aPDI treatment decreased the viable number of *C. sakazakii* in the infant formula with a margin of 85% (Figure 8A), in the studied contamination model. Of note, no pH values of PIF were altered upon treatment (Figure 8B). Next we examined the PIF preservation under various storage conditions. Seen from Figures 8C–F, desiccation or heated stress reduced the contamination of *C. sakazakii* on a case-to-case basis, whereas in all cases their survival rate further declined with an early exposure of aPDI. This data might suggest that aPDI has prophylactic effect on PIF persistence. In summary, aPDI can decontaminate PIF from *C. sakazakii* while maintaining the normal food property.

## Discussion

In the present study, the HB-mediated aPDI was used to reduce the survival of *C. sakazakii*. As a species of opportunistic foodborne pathogen, *C. sakazakii* contaminated powdered infant formula, and caused life-threatening necrotizing enterocolitis and meningitis of neonates (Novotny et al., 2022). Although a variety of natural extracts have been shown to have antibacterial effects on *C. sakazakii* (Chang et al., 2021), to the best of our knowledge, this is the first report to involve aPDI in the decontamination of this ubiquitous pathogen. aPDI prevails over the conventional antimicrobial methods with respect to bacterial sensitivity, strain specificity and visible light usage (Su et al., 2011b; Bartolomeu et al., 2016). As an alternative approach, blue light (405-nm LED) has been successfully applied in the control of *C. sakazakii*, resulting in a reduction of viable cells by 2.5 log (Huang et al., 2020). Despite these encouraging advances, the bactericidal efficacy still needs to be further potentiated. Wu et al. (2021) stated that *C. sakazakii* cells were more resistant to a single intervention of either blue light or 0.02% H_2_O_2_, but the combined intervention attenuated its survival up to 5~6 log CFU. Whilst this approach did not truly take advantage of light irradiation, aPDI solved this issue by adding photosensitizer to the system, triggering photodynamic activity to kill bacteria (3.6 log). Since this result was accomplished under a mild condition, that is, 30 μM HB and 60 J/cm^2^ (or 15 min) illumination, there is still space to further enhance the antimicrobial effect by increasing the PS/light dose.

Gram-negative bacterium is usually considered more resistant to aPDI than gram-positive counterpart. This discrepancy is associated with anatomical features, that is, the presence of outer membrane layer could protect gram-negative cells from dye binding and ROS invasion (Rapacka-Zdonczyk et al., 2021a). When HB was applied to photokill *S. aureus*, 500 nM HB decreased the viable staphylococcal cells by 4~5 log with the aid of 5 J/cm^2^ of blue light (Li et al., 2021). This efficacy is superior to the case of *C. sakazakii*, whereas 3.6 log reduction could only be achieved by 30 μM HB and 60 J/cm^2^ illumination. Thus *C. sakazakii* seems to consist with the intractability of inhibiting gram-negative bacteria with the aPDI-induced pressure. In an unpublished work, we found it very difficult to sterilize *E. coli* O157:H7 by the HB-based aPDI, requiring the synergy of additional antibacterial agents. Compared to the nanomolar dosage used in photokilling *S. aureus* or other gram-positive bacteria, the conditions used here should not be considered very efficient, because the micromolar magnitude of PS has to be applied to drive the bacterial mortality up to 3 logs. The major reason should reside in the intrinsic anatomical features of *C. sakazakii* as a gram-negative bacterium, which also require 60~90 μM of toluidine blue O to achieve the similar outcome in another aPDI instance (Du et al., 2021). This discrepancy between the gram-counterparts was also observed when curcumin was used to photokill *E. coli* and *S. aureus*, wherein the 1.06 ± 0.13 and 2.34 ± 0.13 log reductions were fulfilled, respectively, (Bhavya and Hebbar, 2019). Therefore, the susceptibility against aPDI is highly dependent on the specific microbes under study, and further efforts should be made to search for the utmost optimal photosensitizers to sterilize *C. sakazakii*.

Biofilm is formed by some microbes to resist to environmental stress. It has been reported that *C. sakazakii* can attach to the surface of smooth materials to form biofilm (Borucki et al., 2003). Thus, despite that biofilm is quite unlikely to exist on the dry powder surface, it’s important for us to use biofilm status to assess the *bona fide* resistance of bacterial cells to aPDI, as well as track down the principal mechanistic insight. As evidenced here (Figure 4), the HB-mediated photodynamic action is robust enough to curb the ability of impaired cells to form biofilm, thus aPDI gains an additional advantage of disrupting the biofilm defense of *C. sakazakii*. Besides, it’s noteworthy that the detrimental effect spanned the entire biofilm formation process, which is defined as initial attachment, irreversible attachment, maturation I, maturation II and dispersion (Monroe, 2007). Since the variable stage was characterized by distinct molecular and subcellular action, the exact phase with the utmost susceptibility to aPDI remains unknown and needs to be dissected in future investigations.

CpxRA is an important TCS in some gram-negative bacteria like *E. coli* and *Salmonella*. In terms of *C. sakazakii*, its survival and virulence are closely related to the membrane-bound TCS, as exemplified by YeiE, a LysR-type transcriptional regulator (Hong et al., 2022). As the alternative global regulator, CpxRA was previously shown to regulate the tolerance to osmotic stress in *C. sakazakii* (Andreani et al., 2019). The present study demonstrated roles of CpxRA in the aPDI-mediated impairment. As a global regulatory system, CpxRA is assumed to modulate the expression of multiple genes, including a porin called OmpC. OmpC accounts for the substance transport across outer membrane, which was closely associated with antibiotic resistance and biofilm formation (Gao et al., 2021). Based on the findings here, it’s tempting to speculate that CpxRA-OmpC constitutes a major molecular cascade to mediate the inhibitory effect of aPDI on *C. sakazakii*.

As a regulator, CpxR coupled with CpxA in expressional changes upon aPDI-induced pressure (Figure 4C), which, however, showed discordant orientation in the presence or absence of CpxA knockout. The similar interaction was also reported in a previous study, which stated that CpxA deletion can lead to constitutive activation of the CpxRA system (Jing et al., 2021). This odd performance is likely ascribed to the additional phosphatase activity of CpxA, which mutation caused a reinforced existence of CpxR-P. Meanwhile, CpxR could accept a phosphoryl group from acetyl phosphate as a surrogate to maintain its turnover. The upregulated CpxR then promoted the expression of OmpC in sequence, showing relevance with the entry of ROS or PS. As for another key member of porins, namely OmpF, its alteration was not associated with the regulatory function of CpxRA in the studied context (data not shown). Still, the detailed molecular network leading to CpxR accumulation still remains elusive, and should become the subject of the subsequent studies. In addition, while CpxRA transduction signaling was found to regulate the antioxidative responsiveness of *Salmonella enterica* Serovar Typhimurium (Lopez et al., 2018), no prior report has linked this TCS with aPDI functioning, which might be viewed as an innovative finding of the present study.

Powdered infant formula is not sterile, occasionally containing *C. sakazakii* capable of causing serious illness. Fei et al. (2017) detected *C. sakazakii* contamination in 56 out of 2020 PIF samples, a sign of severity of *C. sakazakii* contamination. Based on its potency in eradicating pathogens in the buffered system, aPDI was then utilized in PIF decontamination, and, as a result, inhibited the growth of *C. sakazakii* (Figure 8). Massive merits are provided by visible light: liberating the autoclave work from spatiotemporal restrictions, as well as constantly attacking the surrounding pathogens. A comparable example is the use of single blue light (Zheng et al., 2021), which showed that 405-nm LED killed *C. sakazakii* in PIF by 0.9 log at a dose of 546 J/cm^2^. The blue light illumination gains an advantage of simplicity and safety, it otherwise did not reach the similar strength as aPDI (60 J/cm^2^). Moreover, when PIF was placed under various conditions like desiccation and high temperatures, a prior treatment of aPDI always accounted for a lower cell number of *C. sakazakii*, thereby qualified as an alternative strategy to decontaminate PIF during storage. Despite its application potential in PIF storage, the issue that cannot be ignored is the safety and acceptance of HB addition to the milk powder. Although HB is long considered safe due to their practice in the fields of Chinese traditional medicine, new regulations were required to clarify its use in the PIF. Alternatively, the incorporation of dye into the soft-package in some cases might offer a promising solution while retaining its antimicrobial activity.

In conclusion, hypocrellin-mediated aPDI is first used to inhibit the growth of *Cronobacter sakazakii*, and found potent in eliminating this prevalent foodborne pathogen. The bactericidal efficacy was achieved by disrupting cell envelope integrity, as well as by deregulating CpxRA-OmpC pathway. The CpxA deletion curbed the antioxidative defense of *C. sakazakii*, underscoring the mediatory importance of CpxRA. Overall, this study demonstrates that aPDI can serve as an alternative and promising approach to decontaminate *Cronobacter sakazakii* from food preservation.

## Data availability statement

The original contributions presented in the study are included in the article/Supplementary material, further inquiries can be directed to the corresponding authors.

## Author contributions

YX and H-LW conceived the project. YX made the concrete directions and wrote the paper. JX and HY carried out most experiments. JX summarized the data and designed the illustrations. YL, QL, XW, and LL helped with mutant construction, aPDI trial, and microscopic graphing. XM and HT participated in data review and provided valuable suggestions. All authors contributed to the article and approved the submitted version.

## Funding

This work was supported by the Bingtuan Science and Technology Program (No. 2022AB004), the Open Project of Anhui Key Laboratory of Tobacco Chemistry (No. 2021307), National Natural Science Foundation of China (Nos. 82073592 and 81773475), Key Technologies Research and Development Program of China (Nos. 2018YFC1602204 and 2018YFC1602201), and Fundamental Research Funds for the Central Universities (JZ2021HGTB0114).

## Conflict of interest

The authors declare that the research was conducted in the absence of any commercial or financial relationships that could be construed as a potential conflict of interest.

## Publisher’s note

All claims expressed in this article are solely those of the authors and do not necessarily represent those of their affiliated organizations, or those of the publisher, the editors and the reviewers. Any product that may be evaluated in this article, or claim that may be made by its manufacturer, is not guaranteed or endorsed by the publisher.

## Supplementary material

The Supplementary material for this article can be found online at: https://www.frontiersin.org/articles/10.3389/fmicb.2022.1063425/full#supplementary-material

SUPPLEMENTARY FIGURE S1 The growth curve of *Cronobacter sakazakii* ATCC29544 and ΔCpxA. Values are the mean ± SEM (standard error of mean) from 3 replicates.

SUPPLEMENTARY FIGURE S2 The inhibitory effect of saline and DMSO on *C. sakazakii*. No photosensitizer was supplemented in this test, and L refers to the illumination with 460 nm-LED. Values are the mean ± SEM (standard error of mean) from 3 replicates. ns, *p* > 0.05.

SUPPLEMENTARY FIGURE S3 ROS stimulated by aPDI on ΔCpxA mutant strain. After DCFH-DA staining, representative microscopy images **(A)** and fluorescence density **(B)** were shown. Scale bar represents 50 μm. Values are the mean ± SEM (standard error of mean) from 3~4 replicates. *p < 634 0.05, **p < 0.01, ***p < 0.001.
