# Supplementary figures and images for: Antimicrobial photodynamic inactivation as an alternative approach to inhibit the growth of Cronobacter sakazakii by fine-tuning the activity of CpxRA two-component system
Source: Front Microbiol. 2023 Jan 17;13:1063425. doi: 10.3389/fmicb.2022.1063425 (PMC9886882; doi:10.3389/fmicb.2022.1063425)

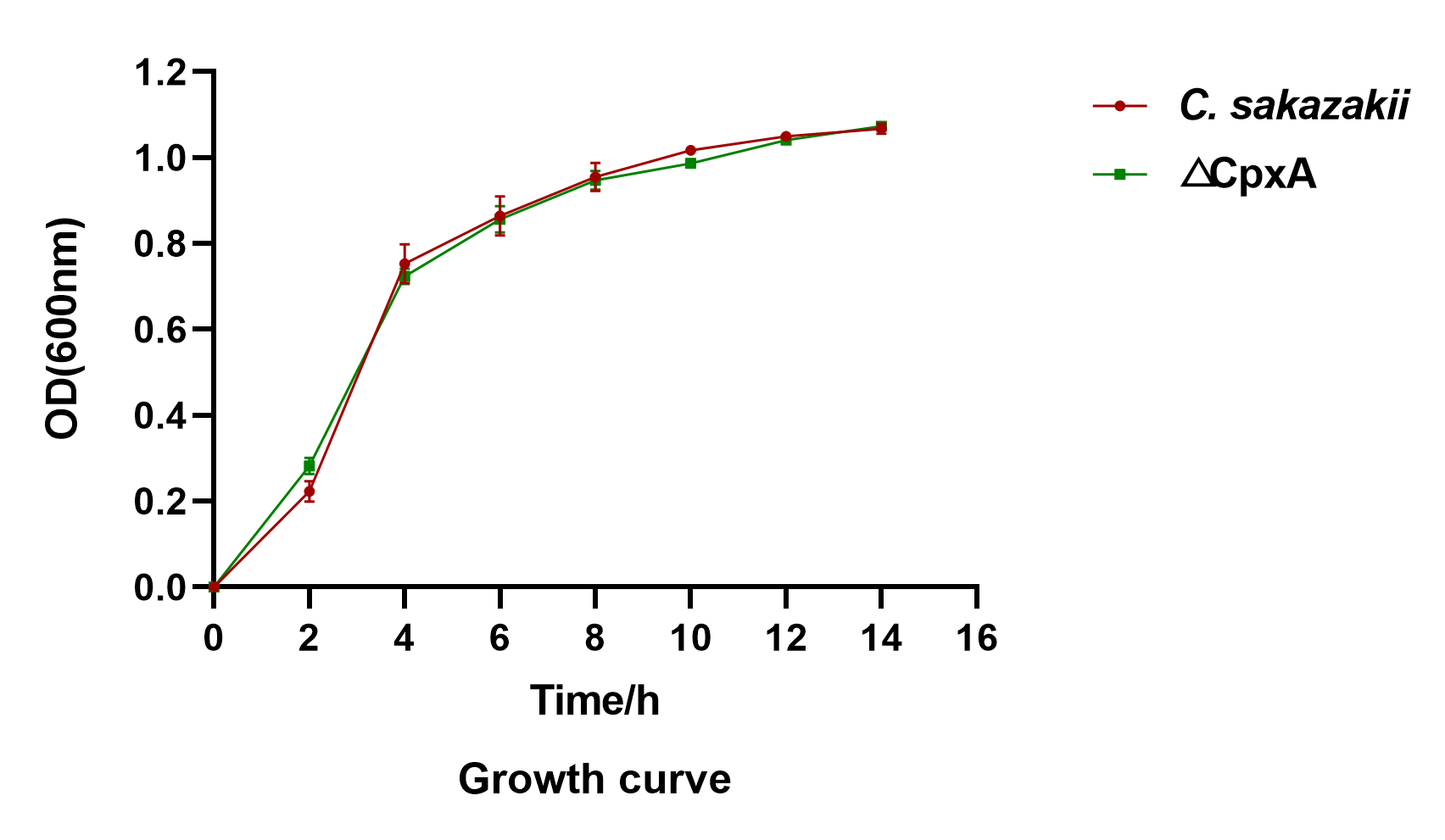

Supplement: SUPPLEMENTARY FIGURE S1 — The growth curve of Cronobacter sakazakii ATCC29544 and ΔCpxA. Values are the mean ± SEM (standard error of mean) from 3 replicates. [file Image_1.TIF]

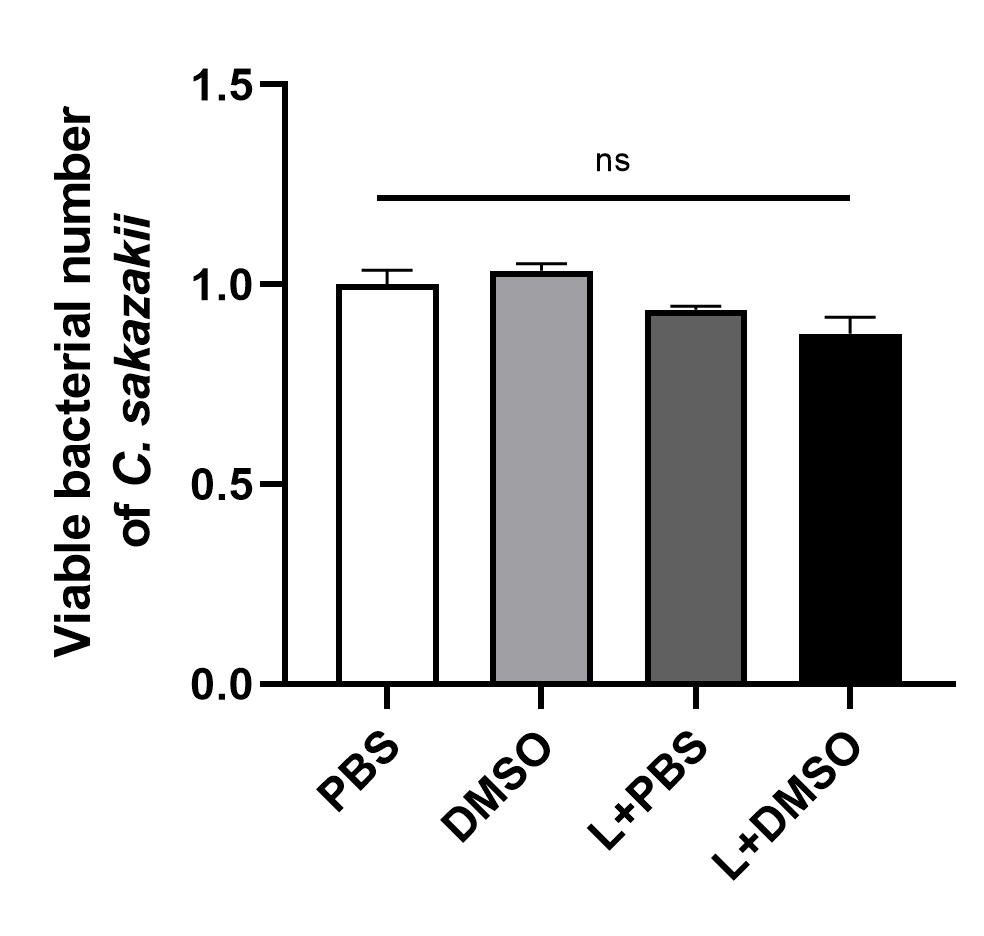

Supplement: SUPPLEMENTARY FIGURE S2 — The inhibitory effect of saline and DMSO on C. sakazakii. No photosensitizer was supplemented in this test, and L refers to the illumination with 460 nm-LED. Values are the mean ± SEM (standard error of mean) from 3 replicates. ns, p > 0.05. [file Image_2.TIF]

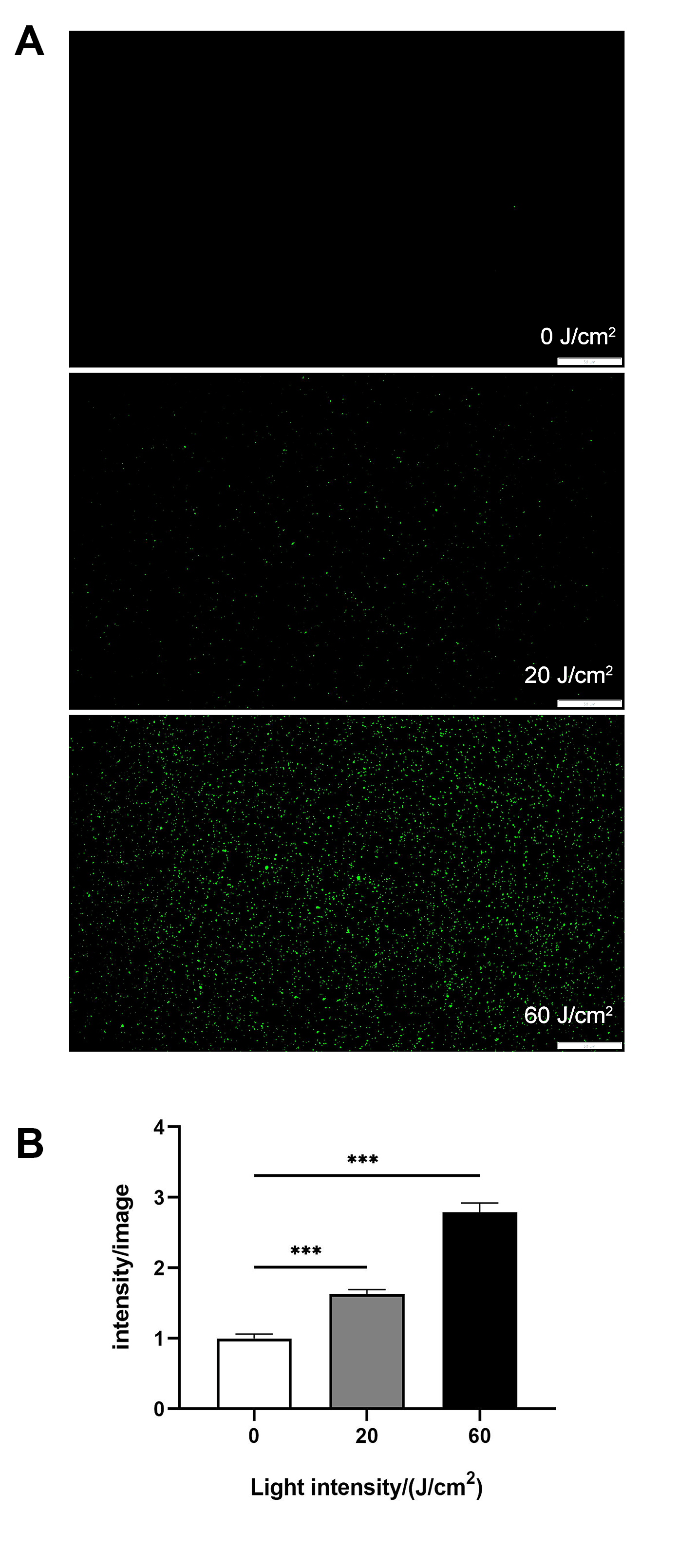

Supplement: SUPPLEMENTARY FIGURE S3 — ROS stimulated by aPDI on ΔCpxA mutant strain. After DCFH-DA staining, representative microscopy images (A) and fluorescence density (B) were shown. Scale bar represents 50 μm. Values are the mean ± SEM (standard error of mean) from 3~4 replicates. *p < 634 0.05, **p < 0.01, ***p < 0.001. [file Image_3.TIF]
